# 4-Bromo-*N*-[4-(diethyl­amino)­benzyl­idene]aniline

**DOI:** 10.1107/S1600536810033726

**Published:** 2010-08-28

**Authors:** Xiao-Fang Li

**Affiliations:** a College of Automation & Electronic Engineering, Qingdao University of Science & Technology, Qingdao 261500, People’s Republic of China

## Abstract

The asymmetric unit of the title compound, C_17_H_19_BrN_2_, contains two independent mol­ecules. The dihedral angles between the two benzene rings in are 60.4 (2) and 61.0 (2)°.

## Related literature

For applications of Schiff base compounds, see: Yu *et al.* (2007 ▶). For related structures, see: You *et al.* (2004 ▶); Yu *et al.* (2007 ▶); Zhang (2010 ▶).
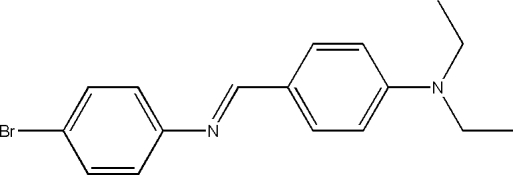


## Experimental

### 

#### Crystal data

C_17_H_19_BrN_2_
*M* *_r_* = 331.25
Triclinic, 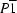

*a* = 10.1863 (11) Å
*b* = 12.3527 (13) Å
*c* = 14.3400 (15) Å
α = 112.936 (2)°
β = 92.986 (1)°
γ = 104.305 (1)°
*V* = 1587.8 (3) Å^3^
*Z* = 4
Mo *K*α radiation
μ = 2.58 mm^−1^
*T* = 298 K
0.45 × 0.39 × 0.38 mm

#### Data collection

Bruker SMART CCD diffractometer
Absorption correction: multi-scan (*SADABS*; Sheldrick, 1996 ▶) *T*_min_ = 0.390, *T*_max_ = 0.440
8347 measured reflections
5530 independent reflections
2795 reflections with *I* > 2σ(*I*)
*R*_int_ = 0.031

#### Refinement

*R*[*F* ^2^ > 2σ(*F* ^2^)] = 0.048
*wR*(*F* ^2^) = 0.115
*S* = 1.02
5530 reflections
365 parameters
H-atom parameters constrained
Δρ_max_ = 0.45 e Å^−3^
Δρ_min_ = −0.55 e Å^−3^

### 

Data collection: *SMART* (Bruker, 1997 ▶); cell refinement: *SAINT* (Bruker, 1997 ▶); data reduction: *SAINT*; program(s) used to solve structure: *SHELXS97* (Sheldrick, 2008 ▶); program(s) used to refine structure: *SHELXL97* (Sheldrick, 2008 ▶); molecular graphics: *SHELXTL* (Sheldrick, 2008 ▶); software used to prepare material for publication: *SHELXTL*.

## Supplementary Material

Crystal structure: contains datablocks global, I. DOI: 10.1107/S1600536810033726/lh5115sup1.cif

Structure factors: contains datablocks I. DOI: 10.1107/S1600536810033726/lh5115Isup2.hkl

Additional supplementary materials:  crystallographic information; 3D view; checkCIF report

## Figures and Tables

**Fig. 1.**
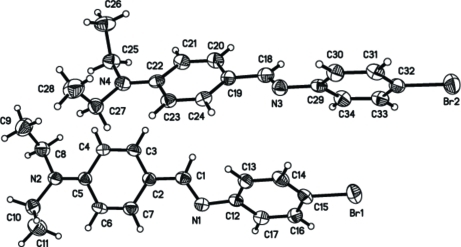
The asymmetric unit of the title compound, drawn with 30% probability ellipsoids.

## supplementary crystallographic information

### Comment

Schiff base compounds have been used as fine chemicals and medical substrates. They are important ligands in coordination chemistry due to their ease of preparation (Yu *et al.*, 2007). In this paper, the crystal structure of the title compound is reported. The asymmetric unit of the title compound contains two independent molecules  (Fig. 1). The dihedral angles between the two benzene rings in each molecule are 60.4 (2) ° and 61.0 (2) °. Bond lengths and angles are comparable to those observed for 4-chloro-*N*-[4-(dimethylamino)benzylidene]aniline (You, *et al.*, 2004) and 4-Chloro-*N*-[4-(diethylamino)benzylidene]aniline (Zhang, 2010).

### Experimental

A mixture of 4-(diethylamino)benzaldehyde (0.01 mol) and 4-bromobenzenamine (0.01 mol) in ethanol (10 ml) was refluxed for 2 h. After cooling, filtration and drying, the title compound was obtained. 10 mg of the title compound was dissolved in 15 ml ethanol, and the solution was kept at room temperature. The single-crystal suitable for X-ray determination was obtained by evaporation from ethanol solution of the title compound after a week.

### Refinement

H atoms were initially located from difference maps and then refined in a riding model with C—H = 0.93–0.97 Å and *U*_iso_(H) = 1.2*U*_eq_(C) or 1.5*U*_eq_(methyl C).

### Crystal data

**Table:**

C_17_H_19_BrN_2_	*Z* = 4
*M**_r_* = 331.25	*F*(000) = 680
Triclinic, *P*1	*D*_x_ = 1.386 Mg m^−^^3^
Hall symbol: -P 1	Mo *K*α radiation, λ = 0.71073 Å
*a* = 10.1863 (11) Å	Cell parameters from 2343 reflections
*b* = 12.3527 (13) Å	θ = 2.6–22.3°
*c* = 14.3400 (15) Å	µ = 2.58 mm^−^^1^
α = 112.936 (2)°	*T* = 298 K
β = 92.986 (1)°	Block, light yellow
γ = 104.305 (1)°	0.45 × 0.39 × 0.38 mm
*V* = 1587.8 (3) Å^3^	

### Data collection

**Table:**

Bruker SMART CCD diffractometer	5530 independent reflections
Radiation source: fine-focus sealed tube	2795 reflections with *I* > 2σ(*I*)
graphite	*R*_int_ = 0.031
Detector resolution: 9 pixels mm^-1^	θ_max_ = 25.0°, θ_min_ = 1.6°
φ and ω scans	*h* = −11→12
Absorption correction: multi-scan (*SADABS*; Sheldrick, 1996)	*k* = −14→14
*T*_min_ = 0.390, *T*_max_ = 0.440	*l* = −17→13
8347 measured reflections	

### Refinement

**Table:**

Refinement on *F*^2^	Primary atom site location: structure-invariant direct methods
Least-squares matrix: full	Secondary atom site location: difference Fourier map
*R*[*F*^2^ > 2σ(*F*^2^)] = 0.048	Hydrogen site location: inferred from neighbouring sites
*wR*(*F*^2^) = 0.115	H-atom parameters constrained
*S* = 1.02	*w* = 1/[σ^2^(*F*_o_^2^) + (0.046*P*)^2^] where *P* = (*F*_o_^2^ + 2*F*_c_^2^)/3
5530 reflections	(Δ/σ)_max_ = 0.001
365 parameters	Δρ_max_ = 0.45 e Å^−^^3^
0 restraints	Δρ_min_ = −0.55 e Å^−^^3^

### Special details

**Table:**

Geometry. All e.s.d.'s (except the e.s.d. in the dihedral angle between two l.s. planes) are estimated using the full covariance matrix. The cell e.s.d.'s are taken into account individually in the estimation of e.s.d.'s in distances, angles and torsion angles; correlations between e.s.d.'s in cell parameters are only used when they are defined by crystal symmetry. An approximate (isotropic) treatment of cell e.s.d.'s is used for estimating e.s.d.'s involving l.s. planes.
Refinement. Refinement of *F*^2^ against ALL reflections. The weighted *R*-factor *wR* and goodness of fit *S* are based on *F*^2^, conventional *R*-factors *R* are based on *F*, with *F* set to zero for negative *F*^2^. The threshold expression of *F*^2^ > σ(*F*^2^) is used only for calculating *R*-factors(gt) *etc*. and is not relevant to the choice of reflections for refinement. *R*-factors based on *F*^2^ are statistically about twice as large as those based on *F*, and *R*- factors based on ALL data will be even larger.

### Fractional atomic coordinates and isotropic or equivalent isotropic displacement parameters (Å^2^)

**Table:**

	*x*	*y*	*z*	*U*_iso_*/*U*_eq_	
Br1	0.63511 (6)	0.55016 (5)	0.91153 (4)	0.0924 (2)	
Br2	0.66046 (6)	0.09334 (5)	0.95086 (4)	0.0763 (2)	
N1	0.3683 (4)	0.6583 (3)	0.5818 (3)	0.0545 (10)	
N2	−0.0254 (4)	0.6568 (3)	0.2131 (3)	0.0618 (10)	
N3	0.3916 (4)	0.1413 (3)	0.5863 (3)	0.0548 (10)	
N4	0.0089 (4)	0.2236 (3)	0.2607 (3)	0.0546 (10)	
C1	0.2611 (5)	0.5804 (4)	0.5184 (3)	0.0527 (12)	
H1	0.2260	0.5062	0.5235	0.063*	
C2	0.1917 (5)	0.6014 (4)	0.4395 (3)	0.0473 (11)	
C3	0.0608 (5)	0.5270 (4)	0.3888 (3)	0.0519 (11)	
H3	0.0204	0.4622	0.4055	0.062*	
C4	−0.0118 (4)	0.5440 (4)	0.3156 (3)	0.0521 (11)	
H4	−0.1001	0.4924	0.2849	0.062*	
C5	0.0457 (5)	0.6385 (4)	0.2864 (3)	0.0497 (11)	
C6	0.1787 (5)	0.7138 (4)	0.3369 (3)	0.0568 (12)	
H6	0.2206	0.7771	0.3189	0.068*	
C7	0.2474 (5)	0.6970 (4)	0.4109 (3)	0.0570 (12)	
H7	0.3342	0.7505	0.4437	0.068*	
C8	−0.1677 (5)	0.5849 (4)	0.1678 (3)	0.0656 (14)	
H8A	−0.2121	0.6319	0.1430	0.079*	
H8B	−0.2156	0.5705	0.2205	0.079*	
C9	−0.1800 (6)	0.4626 (5)	0.0798 (4)	0.0963 (18)	
H9A	−0.1391	0.4763	0.0251	0.144*	
H9B	−0.2753	0.4171	0.0554	0.144*	
H9C	−0.1335	0.4167	0.1033	0.144*	
C10	0.0409 (5)	0.7398 (4)	0.1669 (3)	0.0661 (13)	
H10A	0.0001	0.7054	0.0951	0.079*	
H10B	0.1376	0.7448	0.1702	0.079*	
C11	0.0271 (6)	0.8664 (4)	0.2190 (4)	0.0936 (18)	
H11A	−0.0683	0.8619	0.2198	0.140*	
H11B	0.0650	0.9143	0.1825	0.140*	
H11C	0.0757	0.9045	0.2883	0.140*	
C12	0.4256 (4)	0.6269 (4)	0.6563 (3)	0.0465 (11)	
C13	0.4527 (4)	0.5157 (4)	0.6331 (3)	0.0558 (12)	
H13	0.4283	0.4558	0.5659	0.067*	
C14	0.5154 (5)	0.4918 (4)	0.7080 (3)	0.0608 (13)	
H14	0.5339	0.4170	0.6915	0.073*	
C15	0.5501 (4)	0.5810 (4)	0.8077 (3)	0.0557 (12)	
C16	0.5253 (4)	0.6932 (4)	0.8328 (3)	0.0565 (12)	
H16	0.5484	0.7521	0.9003	0.068*	
C17	0.4664 (4)	0.7169 (4)	0.7578 (3)	0.0533 (12)	
H17	0.4533	0.7936	0.7741	0.064*	
C18	0.2677 (5)	0.1487 (4)	0.5796 (3)	0.0580 (12)	
H18	0.2170	0.1400	0.6296	0.070*	
C19	0.2032 (5)	0.1699 (4)	0.4987 (3)	0.0500 (11)	
C20	0.0633 (5)	0.1592 (4)	0.4867 (3)	0.0579 (12)	
H20	0.0122	0.1399	0.5329	0.070*	
C21	−0.0015 (5)	0.1758 (4)	0.4104 (3)	0.0572 (12)	
H21	−0.0952	0.1675	0.4055	0.069*	
C22	0.0708 (4)	0.2051 (4)	0.3388 (3)	0.0484 (11)	
C23	0.2142 (4)	0.2194 (4)	0.3527 (3)	0.0501 (11)	
H23	0.2670	0.2413	0.3083	0.060*	
C24	0.2755 (4)	0.2016 (4)	0.4297 (3)	0.0508 (11)	
H24	0.3694	0.2111	0.4363	0.061*	
C25	−0.1419 (4)	0.1959 (4)	0.2406 (3)	0.0645 (13)	
H25A	−0.1652	0.2360	0.1986	0.077*	
H25B	−0.1738	0.2303	0.3054	0.077*	
C26	−0.2177 (5)	0.0589 (4)	0.1868 (4)	0.0831 (16)	
H26A	−0.1875	0.0241	0.1222	0.125*	
H26B	−0.3147	0.0477	0.1752	0.125*	
H26C	−0.1984	0.0189	0.2291	0.125*	
C27	0.0858 (5)	0.2632 (4)	0.1898 (3)	0.0614 (13)	
H27A	0.1768	0.3158	0.2263	0.074*	
H27B	0.0396	0.3115	0.1683	0.074*	
C28	0.1001 (6)	0.1581 (5)	0.0958 (4)	0.0974 (18)	
H28A	0.1378	0.1053	0.1163	0.146*	
H28B	0.1602	0.1899	0.0571	0.146*	
H28C	0.0115	0.1123	0.0540	0.146*	
C29	0.4486 (4)	0.1290 (4)	0.6724 (3)	0.0477 (11)	
C30	0.5184 (4)	0.0424 (4)	0.6583 (3)	0.0576 (12)	
H30	0.5236	−0.0093	0.5919	0.069*	
C31	0.5808 (4)	0.0306 (4)	0.7406 (3)	0.0550 (12)	
H31	0.6247	−0.0298	0.7299	0.066*	
C32	0.5762 (4)	0.1106 (4)	0.8388 (3)	0.0515 (11)	
C33	0.5105 (5)	0.1977 (4)	0.8546 (3)	0.0561 (12)	
H33	0.5086	0.2511	0.9212	0.067*	
C34	0.4466 (5)	0.2079 (4)	0.7729 (3)	0.0565 (12)	
H34	0.4016	0.2678	0.7846	0.068*	

### Atomic displacement parameters (Å^2^)

**Table:**

	*U*^11^	*U*^22^	*U*^33^	*U*^12^	*U*^13^	*U*^23^
Br1	0.1166 (6)	0.0896 (5)	0.0824 (4)	0.0230 (4)	−0.0006 (3)	0.0539 (3)
Br2	0.0902 (4)	0.0839 (4)	0.0634 (3)	0.0281 (3)	0.0118 (3)	0.0378 (3)
N1	0.053 (3)	0.051 (2)	0.058 (2)	0.008 (2)	0.009 (2)	0.024 (2)
N2	0.058 (3)	0.057 (3)	0.070 (3)	0.004 (2)	−0.001 (2)	0.035 (2)
N3	0.059 (3)	0.056 (2)	0.050 (2)	0.017 (2)	0.014 (2)	0.0225 (19)
N4	0.048 (3)	0.053 (2)	0.065 (2)	0.016 (2)	0.014 (2)	0.026 (2)
C1	0.056 (3)	0.042 (3)	0.057 (3)	0.013 (3)	0.018 (2)	0.016 (2)
C2	0.050 (3)	0.045 (3)	0.048 (3)	0.015 (3)	0.012 (2)	0.020 (2)
C3	0.059 (3)	0.039 (3)	0.054 (3)	0.007 (3)	0.014 (2)	0.020 (2)
C4	0.050 (3)	0.043 (3)	0.055 (3)	0.001 (2)	0.003 (2)	0.020 (2)
C5	0.055 (3)	0.044 (3)	0.051 (3)	0.015 (3)	0.011 (2)	0.020 (2)
C6	0.052 (3)	0.045 (3)	0.073 (3)	0.002 (3)	0.013 (3)	0.032 (3)
C7	0.046 (3)	0.058 (3)	0.065 (3)	0.009 (3)	0.008 (2)	0.028 (3)
C8	0.062 (4)	0.066 (4)	0.076 (3)	0.020 (3)	0.006 (3)	0.037 (3)
C9	0.111 (5)	0.076 (4)	0.090 (4)	0.023 (4)	−0.017 (3)	0.029 (3)
C10	0.070 (4)	0.069 (4)	0.067 (3)	0.023 (3)	0.011 (3)	0.033 (3)
C11	0.108 (5)	0.078 (4)	0.115 (4)	0.040 (4)	0.038 (4)	0.050 (4)
C12	0.042 (3)	0.046 (3)	0.053 (3)	0.010 (2)	0.014 (2)	0.023 (2)
C13	0.069 (3)	0.048 (3)	0.049 (3)	0.015 (3)	0.015 (2)	0.019 (2)
C14	0.078 (4)	0.048 (3)	0.065 (3)	0.025 (3)	0.027 (3)	0.027 (3)
C15	0.058 (3)	0.061 (3)	0.063 (3)	0.019 (3)	0.021 (2)	0.038 (3)
C16	0.056 (3)	0.058 (3)	0.051 (3)	0.015 (3)	0.013 (2)	0.019 (2)
C17	0.052 (3)	0.048 (3)	0.061 (3)	0.018 (2)	0.018 (2)	0.021 (3)
C18	0.062 (3)	0.053 (3)	0.049 (3)	0.009 (3)	0.016 (3)	0.016 (2)
C19	0.055 (3)	0.048 (3)	0.043 (3)	0.014 (2)	0.014 (2)	0.015 (2)
C20	0.052 (3)	0.065 (3)	0.061 (3)	0.020 (3)	0.029 (2)	0.027 (3)
C21	0.047 (3)	0.065 (3)	0.065 (3)	0.020 (3)	0.024 (3)	0.029 (3)
C22	0.045 (3)	0.037 (3)	0.055 (3)	0.012 (2)	0.009 (2)	0.011 (2)
C23	0.043 (3)	0.051 (3)	0.052 (3)	0.010 (2)	0.015 (2)	0.019 (2)
C24	0.043 (3)	0.048 (3)	0.055 (3)	0.015 (2)	0.010 (2)	0.013 (2)
C25	0.046 (3)	0.065 (4)	0.087 (3)	0.019 (3)	0.010 (3)	0.034 (3)
C26	0.054 (3)	0.066 (4)	0.120 (4)	0.010 (3)	0.004 (3)	0.036 (3)
C27	0.067 (3)	0.061 (3)	0.060 (3)	0.021 (3)	0.009 (3)	0.028 (3)
C28	0.098 (5)	0.089 (4)	0.079 (4)	0.015 (4)	0.029 (3)	0.013 (3)
C29	0.045 (3)	0.042 (3)	0.054 (3)	0.007 (2)	0.017 (2)	0.020 (2)
C30	0.068 (3)	0.057 (3)	0.048 (3)	0.022 (3)	0.023 (2)	0.018 (2)
C31	0.060 (3)	0.050 (3)	0.062 (3)	0.021 (3)	0.024 (2)	0.026 (3)
C32	0.058 (3)	0.044 (3)	0.050 (3)	0.009 (2)	0.010 (2)	0.020 (2)
C33	0.069 (3)	0.044 (3)	0.049 (3)	0.014 (3)	0.023 (2)	0.013 (2)
C34	0.068 (3)	0.047 (3)	0.057 (3)	0.025 (3)	0.023 (2)	0.018 (3)

### Geometric parameters (Å, °)

**Table:**

Br1—C15	1.893 (4)	C14—C15	1.382 (6)
Br2—C32	1.891 (4)	C14—H14	0.9300
N1—C1	1.281 (5)	C15—C16	1.381 (6)
N1—C12	1.414 (5)	C16—C17	1.367 (5)
N2—C5	1.367 (5)	C16—H16	0.9300
N2—C8	1.459 (5)	C17—H17	0.9300
N2—C10	1.476 (5)	C18—C19	1.444 (5)
N3—C18	1.290 (5)	C18—H18	0.9300
N3—C29	1.414 (5)	C19—C24	1.382 (5)
N4—C22	1.378 (5)	C19—C20	1.394 (5)
N4—C27	1.472 (5)	C20—C21	1.356 (5)
N4—C25	1.476 (5)	C20—H20	0.9300
C1—C2	1.443 (5)	C21—C22	1.402 (5)
C1—H1	0.9300	C21—H21	0.9300
C2—C3	1.387 (5)	C22—C23	1.422 (5)
C2—C7	1.399 (6)	C23—C24	1.358 (5)
C3—C4	1.363 (5)	C23—H23	0.9300
C3—H3	0.9300	C24—H24	0.9300
C4—C5	1.397 (5)	C25—C26	1.526 (6)
C4—H4	0.9300	C25—H25A	0.9700
C5—C6	1.405 (6)	C25—H25B	0.9700
C6—C7	1.350 (5)	C26—H26A	0.9600
C6—H6	0.9300	C26—H26B	0.9600
C7—H7	0.9300	C26—H26C	0.9600
C8—C9	1.515 (6)	C27—C28	1.508 (6)
C8—H8A	0.9700	C27—H27A	0.9700
C8—H8B	0.9700	C27—H27B	0.9700
C9—H9A	0.9600	C28—H28A	0.9600
C9—H9B	0.9600	C28—H28B	0.9600
C9—H9C	0.9600	C28—H28C	0.9600
C10—C11	1.494 (6)	C29—C30	1.384 (5)
C10—H10A	0.9700	C29—C34	1.394 (5)
C10—H10B	0.9700	C30—C31	1.384 (5)
C11—H11A	0.9600	C30—H30	0.9300
C11—H11B	0.9600	C31—C32	1.381 (5)
C11—H11C	0.9600	C31—H31	0.9300
C12—C13	1.384 (5)	C32—C33	1.357 (5)
C12—C17	1.405 (5)	C33—C34	1.374 (5)
C13—C14	1.385 (5)	C33—H33	0.9300
C13—H13	0.9300	C34—H34	0.9300
			
C1—N1—C12	118.0 (4)	C15—C16—H16	120.2
C5—N2—C8	121.6 (4)	C16—C17—C12	120.9 (4)
C5—N2—C10	122.2 (4)	C16—C17—H17	119.5
C8—N2—C10	115.9 (3)	C12—C17—H17	119.5
C18—N3—C29	119.3 (4)	N3—C18—C19	123.7 (4)
C22—N4—C27	123.0 (4)	N3—C18—H18	118.2
C22—N4—C25	120.2 (4)	C19—C18—H18	118.2
C27—N4—C25	116.7 (3)	C24—C19—C20	116.5 (4)
N1—C1—C2	123.6 (4)	C24—C19—C18	122.4 (4)
N1—C1—H1	118.2	C20—C19—C18	121.1 (4)
C2—C1—H1	118.2	C21—C20—C19	122.8 (4)
C3—C2—C7	115.8 (4)	C21—C20—H20	118.6
C3—C2—C1	120.5 (4)	C19—C20—H20	118.6
C7—C2—C1	123.7 (4)	C20—C21—C22	120.9 (4)
C4—C3—C2	123.4 (4)	C20—C21—H21	119.6
C4—C3—H3	118.3	C22—C21—H21	119.6
C2—C3—H3	118.3	N4—C22—C21	122.6 (4)
C3—C4—C5	120.3 (4)	N4—C22—C23	121.0 (4)
C3—C4—H4	119.8	C21—C22—C23	116.4 (4)
C5—C4—H4	119.8	C24—C23—C22	121.1 (4)
N2—C5—C4	121.1 (4)	C24—C23—H23	119.5
N2—C5—C6	122.2 (4)	C22—C23—H23	119.5
C4—C5—C6	116.6 (4)	C23—C24—C19	122.3 (4)
C7—C6—C5	122.0 (4)	C23—C24—H24	118.8
C7—C6—H6	119.0	C19—C24—H24	118.8
C5—C6—H6	119.0	N4—C25—C26	113.9 (3)
C6—C7—C2	121.8 (4)	N4—C25—H25A	108.8
C6—C7—H7	119.1	C26—C25—H25A	108.8
C2—C7—H7	119.1	N4—C25—H25B	108.8
N2—C8—C9	112.8 (4)	C26—C25—H25B	108.8
N2—C8—H8A	109.0	H25A—C25—H25B	107.7
C9—C8—H8A	109.0	C25—C26—H26A	109.5
N2—C8—H8B	109.0	C25—C26—H26B	109.5
C9—C8—H8B	109.0	H26A—C26—H26B	109.5
H8A—C8—H8B	107.8	C25—C26—H26C	109.5
C8—C9—H9A	109.5	H26A—C26—H26C	109.5
C8—C9—H9B	109.5	H26B—C26—H26C	109.5
H9A—C9—H9B	109.5	N4—C27—C28	113.6 (4)
C8—C9—H9C	109.5	N4—C27—H27A	108.8
H9A—C9—H9C	109.5	C28—C27—H27A	108.8
H9B—C9—H9C	109.5	N4—C27—H27B	108.8
N2—C10—C11	113.0 (4)	C28—C27—H27B	108.8
N2—C10—H10A	109.0	H27A—C27—H27B	107.7
C11—C10—H10A	109.0	C27—C28—H28A	109.5
N2—C10—H10B	109.0	C27—C28—H28B	109.5
C11—C10—H10B	109.0	H28A—C28—H28B	109.5
H10A—C10—H10B	107.8	C27—C28—H28C	109.5
C10—C11—H11A	109.5	H28A—C28—H28C	109.5
C10—C11—H11B	109.5	H28B—C28—H28C	109.5
H11A—C11—H11B	109.5	C30—C29—C34	117.8 (4)
C10—C11—H11C	109.5	C30—C29—N3	119.7 (4)
H11A—C11—H11C	109.5	C34—C29—N3	122.3 (4)
H11B—C11—H11C	109.5	C29—C30—C31	121.7 (4)
C13—C12—C17	118.3 (4)	C29—C30—H30	119.2
C13—C12—N1	123.5 (4)	C31—C30—H30	119.2
C17—C12—N1	118.1 (4)	C32—C31—C30	118.6 (4)
C12—C13—C14	121.2 (4)	C32—C31—H31	120.7
C12—C13—H13	119.4	C30—C31—H31	120.7
C14—C13—H13	119.4	C33—C32—C31	120.8 (4)
C15—C14—C13	118.9 (4)	C33—C32—Br2	120.8 (3)
C15—C14—H14	120.5	C31—C32—Br2	118.3 (3)
C13—C14—H14	120.5	C32—C33—C34	120.5 (4)
C16—C15—C14	121.1 (4)	C32—C33—H33	119.7
C16—C15—Br1	119.3 (3)	C34—C33—H33	119.7
C14—C15—Br1	119.7 (3)	C33—C34—C29	120.5 (4)
C17—C16—C15	119.5 (4)	C33—C34—H34	119.7
C17—C16—H16	120.2	C29—C34—H34	119.7
			
C12—N1—C1—C2	179.3 (4)	C29—N3—C18—C19	175.5 (4)
N1—C1—C2—C3	−165.6 (4)	N3—C18—C19—C24	−9.6 (7)
N1—C1—C2—C7	12.7 (7)	N3—C18—C19—C20	170.5 (4)
C7—C2—C3—C4	−0.3 (6)	C24—C19—C20—C21	1.5 (6)
C1—C2—C3—C4	178.2 (4)	C18—C19—C20—C21	−178.5 (4)
C2—C3—C4—C5	1.3 (6)	C19—C20—C21—C22	−0.1 (7)
C8—N2—C5—C4	4.7 (6)	C27—N4—C22—C21	175.4 (4)
C10—N2—C5—C4	−168.3 (4)	C25—N4—C22—C21	−8.7 (6)
C8—N2—C5—C6	−174.5 (4)	C27—N4—C22—C23	−2.3 (6)
C10—N2—C5—C6	12.5 (6)	C25—N4—C22—C23	173.6 (4)
C3—C4—C5—N2	179.9 (4)	C20—C21—C22—N4	−179.5 (4)
C3—C4—C5—C6	−0.8 (6)	C20—C21—C22—C23	−1.6 (6)
N2—C5—C6—C7	178.5 (4)	N4—C22—C23—C24	179.8 (4)
C4—C5—C6—C7	−0.7 (6)	C21—C22—C23—C24	1.9 (6)
C5—C6—C7—C2	1.8 (7)	C22—C23—C24—C19	−0.5 (6)
C3—C2—C7—C6	−1.3 (6)	C20—C19—C24—C23	−1.2 (6)
C1—C2—C7—C6	−179.7 (4)	C18—C19—C24—C23	178.8 (4)
C5—N2—C8—C9	−84.0 (5)	C22—N4—C25—C26	−75.2 (5)
C10—N2—C8—C9	89.5 (5)	C27—N4—C25—C26	101.0 (5)
C5—N2—C10—C11	−94.6 (5)	C22—N4—C27—C28	89.2 (5)
C8—N2—C10—C11	92.0 (5)	C25—N4—C27—C28	−86.9 (5)
C1—N1—C12—C13	48.8 (6)	C18—N3—C29—C30	133.9 (4)
C1—N1—C12—C17	−136.0 (4)	C18—N3—C29—C34	−51.2 (6)
C17—C12—C13—C14	1.5 (7)	C34—C29—C30—C31	2.1 (6)
N1—C12—C13—C14	176.7 (4)	N3—C29—C30—C31	177.3 (4)
C12—C13—C14—C15	0.6 (7)	C29—C30—C31—C32	−2.1 (7)
C13—C14—C15—C16	−1.1 (7)	C30—C31—C32—C33	0.9 (7)
C13—C14—C15—Br1	179.7 (3)	C30—C31—C32—Br2	179.6 (3)
C14—C15—C16—C17	−0.5 (7)	C31—C32—C33—C34	0.2 (7)
Br1—C15—C16—C17	178.7 (3)	Br2—C32—C33—C34	−178.4 (3)
C15—C16—C17—C12	2.7 (7)	C32—C33—C34—C29	−0.2 (7)
C13—C12—C17—C16	−3.2 (6)	C30—C29—C34—C33	−0.9 (7)
N1—C12—C17—C16	−178.6 (4)	N3—C29—C34—C33	−176.0 (4)

### Hydrogen-bond geometry (Å, °)

**Table:**

*D*—H···*A*	*D*—H	H···*A*	*D*···*A*	*D*—H···*A*
?—?···?	?	?	?	?
